# Hospital burden, amputation risk, and mortality trends in diabetic foot patients: a retrospective public health analysis

**DOI:** 10.3389/fpubh.2026.1753221

**Published:** 2026-07-15

**Authors:** Xu Zhu, Chao Fang, HongJun Li, YunXia Feng, WeiLi Zhang

**Affiliations:** Wound Repair and Peripheral Vascular Department, Wuhan Integrated Traditional Chinese and Western Medicine Hospital (Wuhan First Hospital), Wuhan, China

**Keywords:** diabetic foot ulcers, hospitalization burden, in-hospital mortality, lower extremity amputation, retrospective public health analysis

## Abstract

**Background:**

Diabetic foot disease remains one of the leading preventable causes of hospitalization, limb loss, and premature mortality. It is important to understand temporal changes in disease severity and outcomes in order to establish gaps in prevention and acute management. The present study reported on the hospital burden, amputation patterns, and mortality trends of diabetic foot patients over a decade.

**Methods:**

This is a retrospective analysis of 500 patients admitted for diabetic foot complications from 2015 to 2024. The records were reviewed for demographic profile, ulcer grade, severity of infection, co-morbidities, length of stay, surgical interventions, and mortality. The amputation was classified into minor or major, while mortality was analyzed at two stages: during hospitalization and within 30 days. Annual trends were studied to assess the changes in clinical presentation and outcomes.

**Results:**

Diabetic foot complication admissions gradually increased year by year, with later years recording more severe grades of ulcers and high infection burden. Major amputations formed a consistent proportion of the surgical cases, though a slight decline was recorded after 2021. Overall mortality remained similar throughout the study period but was higher among those presenting with sepsis, advanced peripheral arterial disease, or chronic renal impairment. The hospital burden remained high, reflected in prolonged lengths of stay and multiple readmissions.

**Conclusion:**

The 10-year pattern demonstrates persistent clinical severity at presentation, continued reliance on major amputations, and stable but meaningful mortality in diabetic foot patients. The findings stress the need for stronger preventive care, earlier referral, and community-level screening strategies to reduce advanced disease and improve survival.

## 1 Introduction

Hospitalization due to diabetic foot complications reflects not only the acute clinical challenges but also highlights disparities in healthcare resource allocation, as shown in regional studies linking avoidable admissions to primary healthcare capacity (1, 2). Meanwhile, emerging research on molecular markers and novel pathological mechanisms in diabetes offers potential avenues for early detection and targeted treatment, complementing clinical efforts to reduce the high amputation risk and mortality associated with diabetic foot ulcers (3, 4).

The burden of diabetic foot complications on healthcare systems has escalated significantly over recent years, reflecting their critical impact on hospital resources and public health. Diabetic foot ulcers (DFUs) are among the leading causes of hospitalization for patients with diabetes, accounting for a substantial share of inpatient care and economic expenditure (5). Hospital admissions due to diabetic foot issues are driven largely by infections, gangrene, and the need for surgical interventions such as amputations, which are often preceded by prolonged and intensive treatments. This clinical scenario contributes to increased hospital stay durations, elevated costs, and a higher utilization of healthcare resources (6).

Amputation risk in diabetic foot patients remains a central concern, as it not only signifies severe disease progression but also results in considerable morbidity and mortality. Studies show that infections and ischemia are major determinants of amputation, with higher rates observed in those with persistent neuropathy and peripheral vascular disease (7). Despite advances in wound care and revascularization techniques, the incidence of both minor and major amputations has not declined proportionately, highlighting ongoing challenges in early detection and preventative strategies. Hospital costs associated with amputations also constitute a significant part of the overall economic burden, emphasizing the need for improved management protocols (8).

Mortality among patients with diabetic foot ulcers is markedly higher compared to the general diabetic population, often influenced by systemic infections, cardiovascular comorbidities, and the extent of limb ischemia (9). Recent trends reveal that mortality rates remain high, with certain studies indicating that nearly one in six patients may die within a year of hospitalization for foot complications (10). The extent of systemic involvement, coupled with recurrent infections and complications like osteomyelitis, contributes to poor survival outcomes. These trends underscore the importance of comprehensive care approaches that address both local foot health and systemic health risks (11, 12).

Peripheral arterial occlusive disease and osteomyelitis have been well established as significant contributors to lower extremity amputation in patients with diabetic foot disease, but few longitudinal hospital-based studies have comprehensively examined factors associated with hospitalization burden, changes over time in amputation trends, mortality, and the impact of vascular and infectious complications on a single cohort. The current study builds on the previous studies by offering a detailed 10-year assessment of the diabetic foot admissions in a tertiary care hospital with detailed clinical characteristics, treatment modalities and outcomes. This study does not attempt to identify known risk factors, but rather defines the changing spectrum of disease severity and groups of patients most likely to have poor outcomes, which may inform hospital resource planning, multidisciplinary management, and inform future prevention strategies.

This retrospective analysis aims to comprehensively evaluate the evolving patterns of hospital burden, amputation risk, and mortality in diabetic foot patients, integrating data across multiple regions and healthcare settings. The focus is to identify underlying factors driving these trends and to inform evidence-based public health strategies. The study’s objective is to fill existing research gaps by providing a holistic overview of clinical outcomes and healthcare costs over the past decade, offering insights into potential areas for targeted intervention and resource allocation. Although the association of diabetic foot complications with amputation and mortality has been extensively reported, longitudinal evaluations integrating hospitalization burden, amputation patterns, and short-term mortality outcomes within a single cohort remain comparatively limited. The present study contributes contemporary real-world evidence regarding evolving clinical trends over a 10-year observation period.

## 2 Methodology

### 2.1 Study design and setting

This retrospective observational study reviewed electronic medical records of patients admitted with diabetic foot complications at a tertiary care hospital between January 2015 and December 2024. Retrospective designs are commonly used to evaluate long-term outcomes and temporal shifts in diabetic foot care within real-world clinical settings.

### 2.2 Study population and eligibility criteria

The present study is a retrospective study of patients who were admitted with diabetic foot complications from January 2015 to December 2024. A total of 700 medical records were reviewed to be eligible during the study period. Patients included in the study were those who were ≥18 years of age and with a known history of diabetes mellitus, either type 1 or type 2, and had one of the following complications: diabetic foot ulceration, diabetic infection, diabetic ischemic gangrene, or diabetic mixed foot disease. The severity of the ulcer was determined by the Wagner or UT classification systems, both of which have been widely adopted for the evaluation of ulcer depth, ulcer infection and ischemia for studies on the diabetic foot (13, 14).

**Figure 1:**
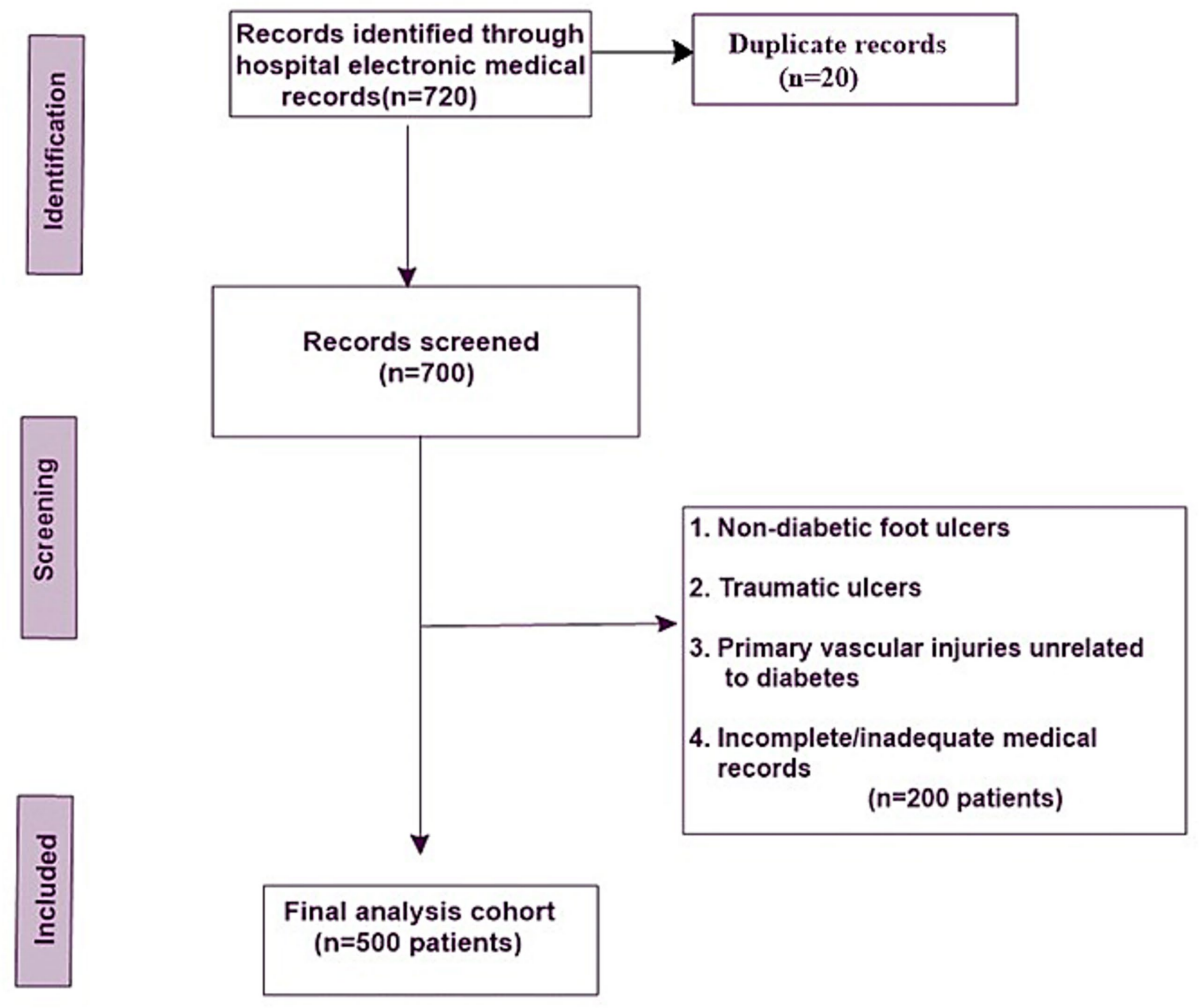
Flow chart illustrating the selection of participant selection for retrospective cohort study.

Patients with traumatic non-diabetic foot ulcers (*n* = 82), incomplete clinical documentation, primary vascular pathologies not associated with diabetes (*n* = 43) and duplicate and follow-up admissions that did not meet the pre-defined eligibility criteria (*n* = 14) were excluded from the records. After selecting the inclusion and exclusion criteria, 500 patients were included in the final analysis. The participant screening and selection process is presented in Figure 1.

### 2.3 Data collection and variables

Data were extracted from electronic health records by trained clinical researchers using a standardized data collection form. Variables included demographic characteristics, diabetes duration, comorbidities, ulcer grade, infection severity, osteomyelitis status, peripheral arterial occlusive disease (PAOD), laboratory parameters, imaging findings, treatment modalities, surgical interventions, and clinical outcomes. Information regarding hospitalization burden, length of stay, readmissions, amputation procedures, and mortality outcomes was also collected. Data extraction was independently verified by senior investigators to ensure accuracy and consistency of the recorded information (15–18).

#### 2.3.1 Clinical definitions

##### 2.3.1.1 Osteomyelitis

The diagnosis of osteomyelitis was made based on the combined clinical, radiological, microbiological and intraoperative findings, using a set of criteria for the diagnosis of diabetic foot infections. Osteomyelitis cases were considered when bone involvement was demonstrated by either plain radiograph or magnetic resonance imaging (MRI) and/or bone culture was positive, or when the diagnosis was confirmed by histopathologic examination or intraoperatively. Those who were diagnosed according to the international criteria of Diabetic Foot Osteomyelitis were included in this group.

##### 2.3.1.2 Peripheral arterial occlusive disease (PAOD)

Peripheral arterial occlusive disease (PAOD) was defined as an atherosclerotic blockage of the arteries in the lower legs that prevented adequate peripheral circulation (19). Patients were identified as having PAOD if objective evidence of arterial insufficiency was obtained in any of the following vascular assessment modalities: ankle-brachial index (ABI) < 0.90, doppler ultrasonography showing significant arterial stenosis or occlusion, computed tomography angiography, digital subtraction angiography or a documented diagnosis by a vascular specialist. When multiple vascular investigations were available, the final diagnosis was established according to the overall clinical assessment documented in the medical records.

When vascular data was available, PAOD was considered mild, moderate, or severe based on ABI values, imaging findings and on the presence of CLI. Patients with PAOD that were not fully documented were excluded from severity-stratified analyses but included in the overall PAOD category.

#### 2.3.2 Outcome categorization

Amputations were defined as minor (toe, ray or forefoot) or major (below knee or above knee). The outcomes of mortality included in-hospital mortality as well as 30-day all-cause mortality after discharge. Thirty-day mortality was defined as death that occurred within 30 days of the index hospitalization. Time till death or 30 days was considered as survival time. Survival analyses (15–18) censored those patients who were not dead at the end of the follow-up period on day 30.

### 2.4 Outcome measures

The main endpoints of interest were hospital burden, amputation risk and trends in mortality throughout the study duration. The burden in the hospital was evaluated through admission rates (per year), hospital day, intensive care unit utilization and readmission rates. Amputations were assessed by the presence of minor amputations and major amputations of the lower extremity. Mortality outcomes consisted of in-hospital mortality and 30-day all-cause mortality. Secondary outcomes were association of major clinical comorbidities with amputation outcomes and mortality outcomes, such as chronic kidney disease, sepsis, osteomyelitis and peripheral arterial occlusive disease (20). The measures were chosen due to their proven clinical significance in DFD and known link to the adverse outcomes of patients (16).

### 2.5 Statistical analysis

Continuous variables were summarized using means and standard deviations or medians with interquartile ranges depending on data distribution. Categorical variables were analyzed using frequencies and percentages. Temporal trends from 2015 to 2024 were examined using chi-square tests for categorical data and linear regression for continuous variables. Multivariable logistic regression was used to identify predictors of major amputation and mortality. Statistical significance was defined as *p* < 0.05. Analytical methods applied here are consistent with standard approaches in diabetic foot epidemiology. The data were statistically analyzed after missing data were assessed. Multiple imputation with chained equations was used for variables with <5% missing data. The imputed and complete-case models did not differ significantly in the results of the sensitivity analyses. Multivariable analyses were not performed for variables that had significant missing data. Linear regression models were used to assess temporal trends for continuous variables while Cochran-Armitage trend tests were used for categorical variables. ANs, amputation rates, and mortality outcomes were quantified using regression coefficients (β), corresponding 95% confidence intervals (CIs), and *p*-values.

## 3 Results

**Figure 2:**
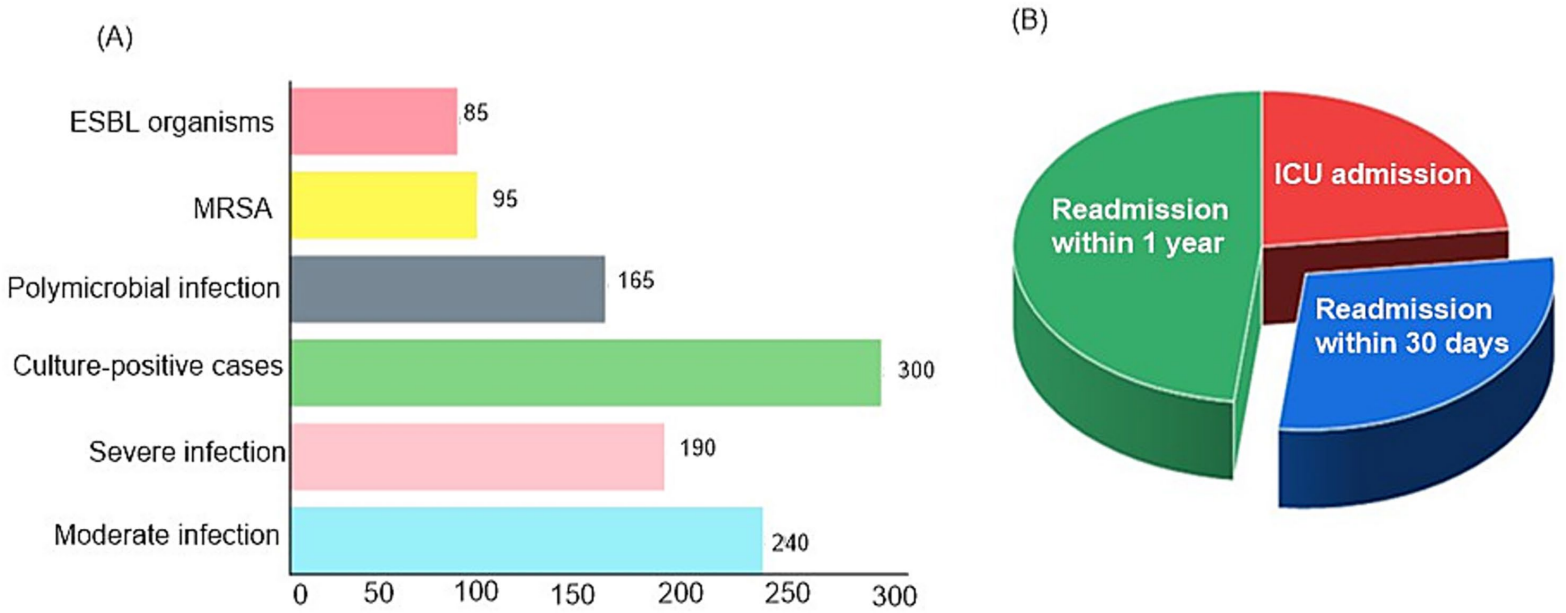
**(A)** Annual trends in diabetic foot admissions, minor amputations, and major amputations from 2015 to 2024. **(B)** Distribution of Wagner ulcer grades among 500 diabetic foot patients.

Infection severity data show that 48 percent presented with moderate infection and 38 percent with severe infection, and cultures were positive in 60 percent of cases, with polymicrobial patterns in 32 percent (Supplementary Table S2). Laboratory parameters indicated systemic inflammation and metabolic stress, with a mean WBC count of 15.2 × 10^9^/L, median CRP of 112 mg/L, and ESR of 73 mm/h (Supplementary Table S3). Ulcer characteristics varied, with 28 percent presenting with Wagner Grade 1–2, 33 percent with Grade 3, and 39 percent with Grade 4–5 lesions (Table 1). Osteomyelitis was documented in 42 percent, peripheral arterial disease in 38 percent, and cellulitis in 66 percent of the cohort. Sepsis was present in 19 percent at admission (Figure 2).

**Table 1:** Clinical presentation and ulcer characteristics.

Variable	Duration (days)	*n* (%)
Ulcer duration before admission	21 (IQR 10–40)	
Wagner Grade 1–2		140 (28.0)
Wagner Grade 3		165 (33.0)
Wagner Grade 4–5		195 (39.0)
Presence of cellulitis		330 (66.0)
Osteomyelitis confirmed (X-ray/MRI)		210 (42.0)
Peripheral arterial disease		190 (38.0)
Sepsis on admission		95 (19.0)

### 3.1 Hospital burden analysis

The median length of stay was 9 days (IQR 6–14), as summarized in Table 2. ICU care was required for 13 percent of patients. Surgical demand was high, with 76 percent undergoing debridement and 49 percent requiring an amputation procedure when minor and major events were combined (Table 3). The burden of minor and major amputation subtypes is detailed in Supplementary Table S4, where toe amputations accounted for 21 percent of all procedures and below-knee amputations for 14 percent. Formal trend analysis demonstrated a significant annual increase in hospital admissions (*β* = 2.03 admissions/year, *p* = 0.004). In contrast, major amputation rates showed a significant downward trend during the observation period (*β* = −0.82% per year, *p* = 0.031), whereas mortality rates remained statistically stable (*β* = −0.07% per year, *p* = 0.641).

**Table 2:** Hospital burden indicators.

Variable	Value	*n* (%)
	Days	
Median length of stay	9 (IQR 6–14)	
ICU admission		65 (13.0)
Readmission within 30 days		80 (16.0)
Readmission within 1 year		135 (27.0)

**Table 3:** Treatment approaches and surgical interventions.

Variable	Value
	*n* (%)
Intravenous antibiotics,	500 (100)
Surgical debridement, *n* (%)	380 (76.0)
Minor amputation (toe/forefoot), *n* (%)	155 (31.0)
Major amputation (BKA/AKA), *n* (%)	90 (18.0)
Revascularization procedure, *n* (%)	40 (8.0)
Negative-pressure wound therapy, *n* (%)	60 (12.0)

Readmissions were frequent, with 16 percent returning within 30 days and 27 percent experiencing a readmission within 1 year (Table 2). Supplementary Table S5 shows that most early readmissions were triggered by infection or wound failure, while one-year readmissions were mostly linked to recurrent infection or development of new ulcers. Trends over time indicate a steady rise in annual admissions from 2015 to 2022, followed by modest declines in 2023 and 2024 (Table 4).

**Table 4:** Annual trends in admissions, amputation, and mortality (2015–2024).

Year	Admissions	Minor amputation (%)	Major amputation (%)	In-hospital mortality (%)
2015	38	30.0	22.0	6.0
2016	42	29.0	23.0	6.5
2017	45	31.0	20.0	7.0
2018	48	30.0	19.0	6.8
2019	52	33.0	18.0	7.2
2020	55	34.0	20.0	8.0
2021	58	33.0	17.0	7.5
2022	62	32.0	16.0	7.0
2023	50	30.0	15.0	6.5
2024	50	29.0	14.0	6.2

### 3.2 Cohort description

**Figure 3:**
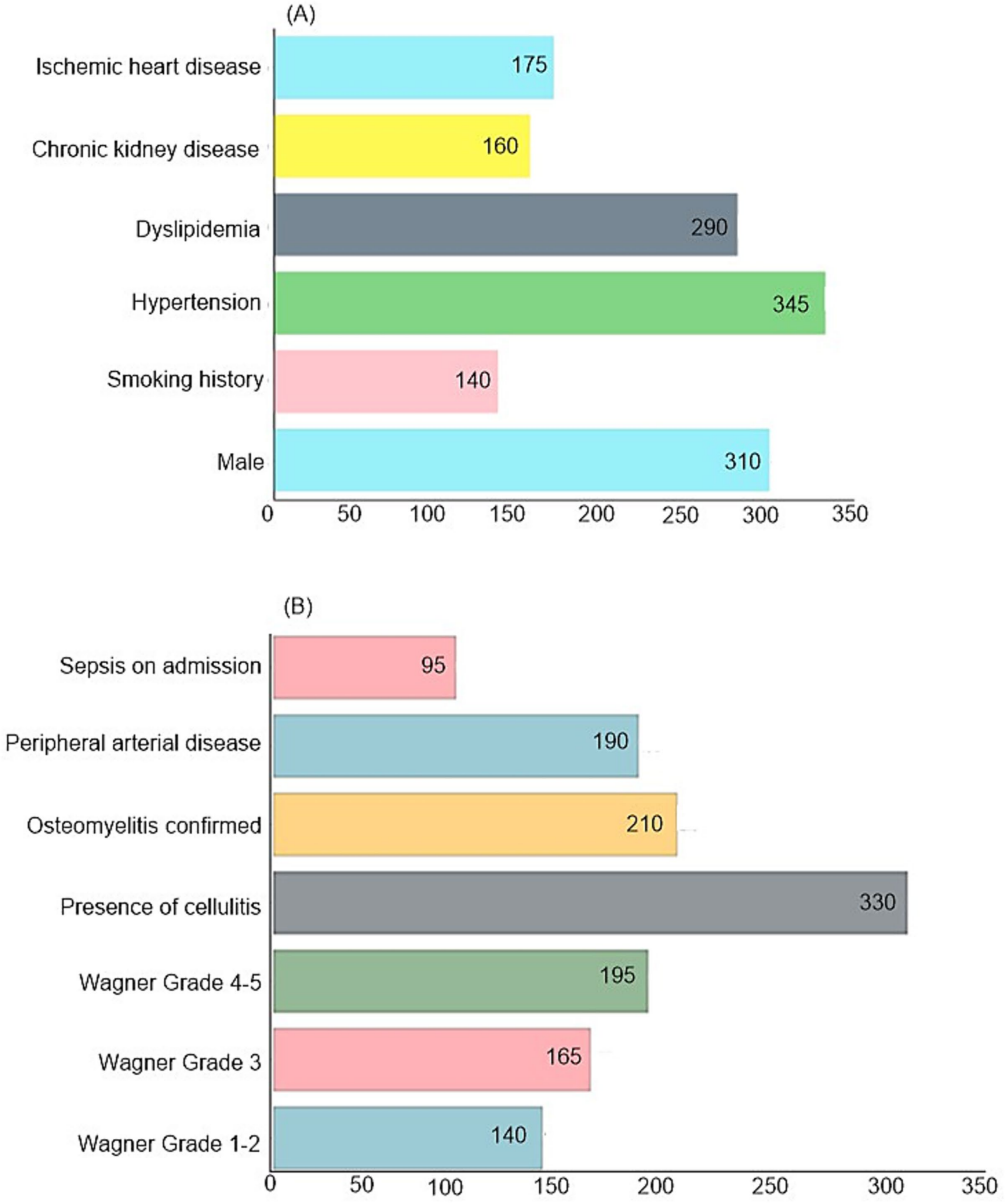
**(A)** Microbial spectrum isolated from infected diabetic foot ulcers. **(B)** Length-of-stay distribution among hospitalized diabetic foot patients.

The study included 500 patients with diabetic foot disease after applying the selection criteria. The cohort had a mean age of 57.8 ± 10.6 years, and men comprised 62 percent of the population. The median duration of diabetes was 12 years (IQR 8–18), and comorbidities were frequent, with hypertension in 69 percent, dyslipidemia in 58 percent, ischemic heart disease in 35 percent, and chronic kidney disease in 32 percent (Table 5). The comorbidity profile in Supplementary Table S1 shows high rates of peripheral neuropathy (83 percent), retinopathy (46 percent), and previous stroke (11 percent) (Figure 3).

**Table 5:** Baseline characteristics of the study population (*N* = 500).

Variable	Value	*n* (%)
	mean ± SD (years)	
Age	57.8 ± 10.6	
Male		310 (62.0)
Duration of diabetes, median	12 (IQR 8–18)	
HbA1c	9.2 ± 1.6	
Smoking history		140 (28.0)
Hypertension		345 (69.0)
Dyslipidemia		290 (58.0)
Chronic kidney disease		160 (32.0)
Ischemic heart disease		175 (35.0)

### 3.3 Amputation trends

**Figure 4:**
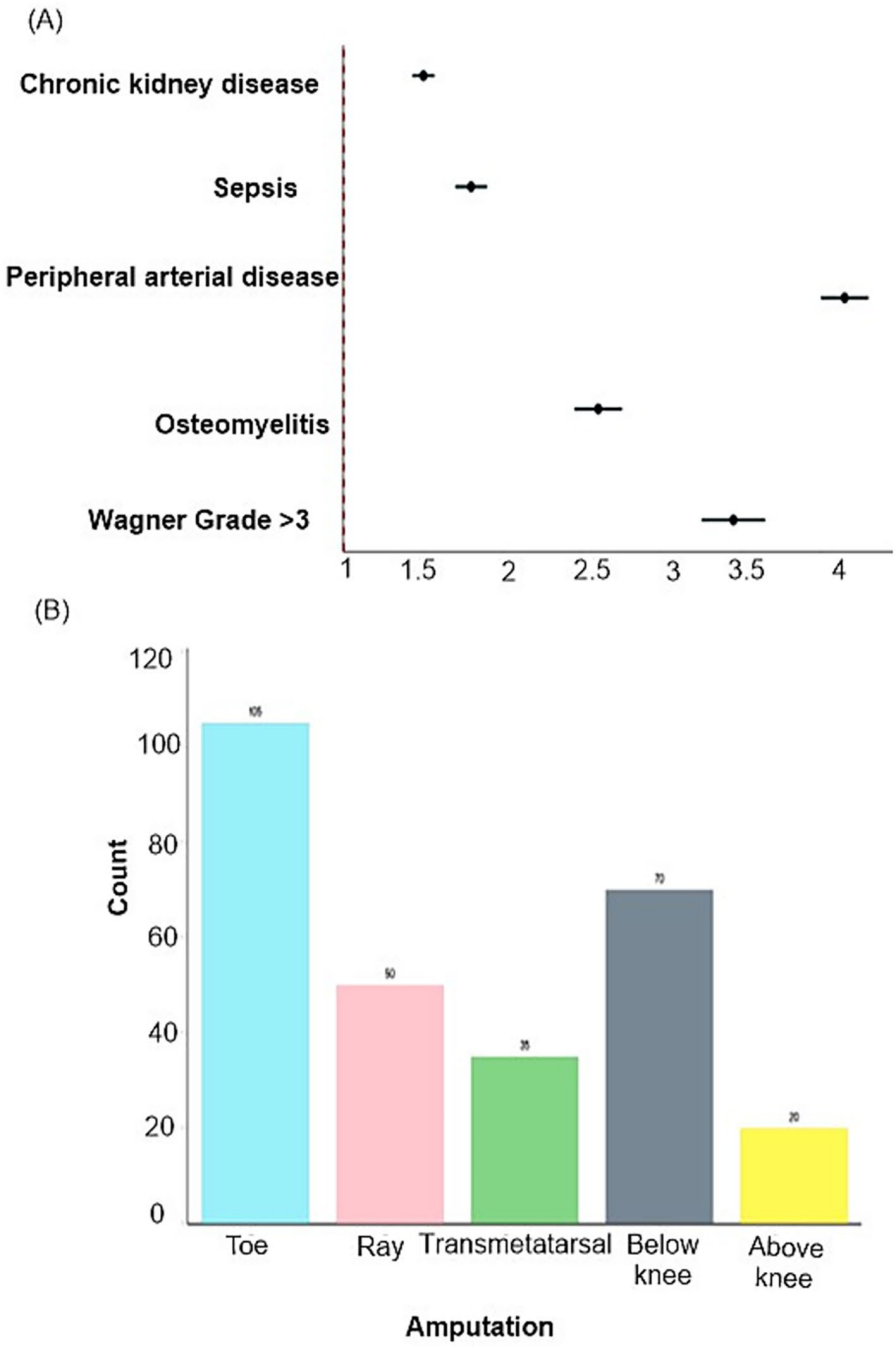
**(A)** Forest plot for major amputation risk predictors in patients with diabetic foot ulcers. **(B)** Breakdown of minor (toe, ray, and transmetatarsal) versus major (below-knee, above-knee) amputation procedures performed in the diabetic foot ulcer patient cohort.

Out of the total cohort, 31 percent underwent minor amputation and 18 percent underwent major amputation. The procedural distribution is presented in Supplementary Table S4 and Figure 4B. Across the study period, the annual proportion of major amputations declined gradually from 22 percent in 2015 to 14 percent in 2024 (Table 4). Minor amputation rates fluctuated between 29 and 34 percent but showed no strong directional trend. Higher-grade ulcers and perfusion deficits showed strong associations with major amputation. Wagner Grade 4–5 lesions accounted for a large share of major procedures, and patients with peripheral arterial disease had a major amputation rate that aligned with the strongest predictor effect (Table 6). Osteomyelitis also contributed meaningfully to the risk profile.

**Table 6:** Amputation predictors: multivariable logistic regression.

Predictor	Adjusted OR	95% CI	*p*-value
Wagner Grade ≥3	3.45	1.90–6.25	<0.001
Osteomyelitis	2.60	1.45–4.65	<0.001
Peripheral arterial disease	4.15	2.10–8.10	<0.001
Sepsis	1.80	1.05–3.10	0.032
Chronic kidney disease	1.50	0.93–2.42	0.080

Multivariable logistic regression confirmed that peripheral arterial disease was the strongest independent predictor of major amputation (adjusted OR 4.15, 95% CI 2.10–8.10, *p* < 0.001), followed by Wagner Grade ≥3 lesions (adjusted OR 3.45, 95% CI 1.90–6.25, *p* < 0.001). Osteomyelitis was also independently associated with increased amputation risk (adjusted OR 2.60, 95% CI 1.45–4.65, *p* < 0.001), while sepsis showed a moderate but significant association (adjusted OR 1.80, 95% CI 1.05–3.10, *p* = 0.032). Chronic kidney disease did not reach statistical significance in the adjusted model (adjusted OR 1.50, 95% CI 0.93–2.42, *p* = 0.080). A comparison of early versus delayed surgery demonstrated an association between earlier surgical intervention and lower frequencies of major amputation; however, causality cannot be inferred because of the retrospective observational design. This aligns with the distribution pattern observed across clinical severity categories (Figure 4A).

### 3.4 Mortality trends

**Figure 5:**
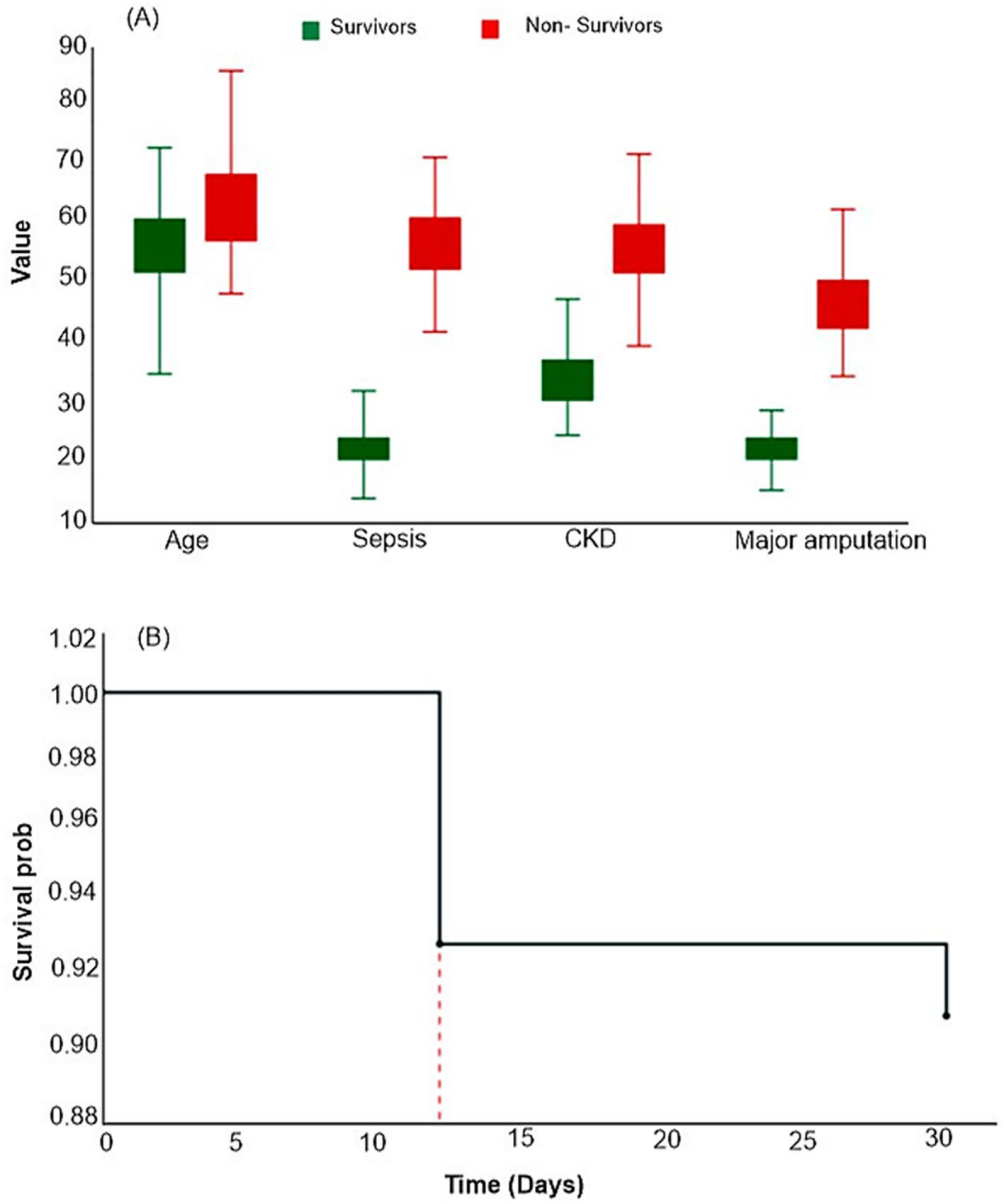
**(A)** Comparison of clinical predictors between survivors (green) and non-survivors (red) in the diabetic foot ulcer cohort. **(B)** Kaplan–Meier survival curve for 30-day mortality among diabetic foot patients.

Mortality was strongly linked to systemic illness at admission. Supplementary Table S6 shows that non-survivors were older (64.4 ± 9.5 vs. 56.5 ± 10.0 years, *p* < 0.001) and had higher rates of sepsis (55.6 percent) and chronic kidney disease (55.6 percent) (Figure 5A). In-hospital mortality was 7 percent, and 30-day mortality reached 9 percent (Supplementary Table S7). Annual mortality rates ranged from 6.0 to 8.0 percent across the study period, with small variations that did not indicate a sustained upward or downward pattern (Figure 5B). Major amputation was also more common among non-survivors.

Multivariable logistic regression identified sepsis as the strongest independent predictor of mortality (adjusted OR 3.80, 95% CI 2.05–7.05, *p* < 0.001). Age ≥65 years was significantly associated with increased mortality risk (adjusted OR 2.30, 95% CI 1.25–4.25, *p* = 0.011), along with chronic kidney disease (adjusted OR 2.95, 95% CI 1.45–6.00, *p* = 0.002) and peripheral arterial disease (adjusted OR 2.10, 95% CI 1.12–3.95, *p* = 0.020). Major amputation showed a positive but non-significant association with mortality (adjusted OR 1.60, 95% CI 0.92–2.80, *p* = 0.090). The median time to death was 12 days (IQR 7–18). Multivariable modeling identified age ≥65 years, sepsis, chronic kidney disease, and peripheral arterial disease as independent predictors of mortality, with adjusted odds ratios ranging from 2.10 to 3.80 (Table 7).

**Table 7:** Mortality predictors: multivariable logistic regression.

Predictor	Adjusted OR	95% CI	*p*-value
Age ≥ 65 years	2.30	1.25–4.25	0.011
Sepsis	3.80	2.05–7.05	<0.001
Chronic kidney disease	2.95	1.45–6.00	0.002
Peripheral arterial disease	2.10	1.12–3.95	0.020
Major amputation	1.60	0.92–2.80	0.090

### 3.5 Risk predictor analysis

Univariable patterns were consistent with the burden of advanced ulceration and systemic illness. In the adjusted model, Wagner Grade ≥3 showed an adjusted odds ratio of 3.45, while peripheral arterial disease had the strongest association with major amputation (adjusted OR 4.15) (Table 6). Osteomyelitis contributed significantly (adjusted OR 2.60), and sepsis also increased risk. The mortality model showed similar predictors, where sepsis remained the most powerful contributor (adjusted OR 3.80). Chronic kidney disease and advanced age also carried significant effect sizes (Table 7). These combined patterns identified a high-risk phenotype characterized by older age, perfusion deficits, systemic inflammation, and advanced tissue destruction.

### 3.6 Antibiotic use patterns

Among the diabetic foot ulcer patient cohort, antibiotic usage was distributed as follows: broad-spectrum beta-lactams were administered to 330 patients (66.0%), glycopeptides to 95 patients (19.0%), carbapenems to 70 patients (14.0%), and step-down oral antibiotics to 420 patients (84.0%). The majority of patients received step-down oral therapy, while broad-spectrum beta-lactams constituted the most common intravenous class. Use of advanced antibiotics such as glycopeptides and carbapenems was less frequent, reflecting their targeted application for resistant or severe infections.

### 3.7 Public health implications

The high rates of surgical intervention, prolonged hospital stays, and recurrent admissions indicate that diabetic foot disease continues to place significant pressure on inpatient services. The median stay of 9 days and ICU utilization of 13 percent reflect sustained resource demands. When combined with an amputation rate approaching 50 percent and mortality approaching 9 percent at 30 days, the overall burden becomes substantial for tertiary-care systems. The observed burden on healthcare services is reflected by prolonged hospital stays, repeated admissions, and frequent surgical procedures. Although formal economic analyses were not performed, these findings suggest substantial healthcare resource utilization. The 10-year pattern observed in Table 4 suggests that, without community-level prevention, the trajectory of admissions will continue to rise. These results support scaling early screening programs, strengthening multidisciplinary foot care pathways, and improving rapid vascular assessment capacity. The sustained readmission burden, recurrent ulceration, and high infection rates reinforce the need for coordinated surveillance and structured foot-care education programs at the community level.

## 4 Discussion

This 10-year cohort offers a clear clinical and prognostic profile of 500 patients hospitalized with diabetic foot disease. Table 5 indicates a population with substantial metabolic and cardiovascular strain, reflected by a mean age of 57.8 years and the familiar male predominance linked to neuropathy and occupational stressors. A median diabetes duration of 12 years matches the expected timeline for microvascular and macrovascular injury, consistent with the high rates of neuropathy, PAD, and CKD seen throughout the dataset. Poor glycemic control is evident from a mean HbA1c of 9.2%, creating conditions that impair healing and intensify infection severity. Hypertension, dyslipidemia, ischemic heart disease, and a notable history of smoking further undermine vascular integrity and help explain the high need for advanced wound care and surgical management observed in the cohort (14, 21).

This is the first study to evaluate the burden of hospitalization, amputation outcomes and short-term mortality in a single tertiary care diabetic foot cohort for a continuous 10-year period. While prior studies have independently assessed vascular disease, infection and/or amputation, a few studies have described how these factors change over time with each other and also added clinical severity, microbiological data, therapy patterns and hospital utilization. This wider viewpoint offers a detailed understanding of how a patient with DFD presents in the hospital setting and what could be done earlier in the course of treatment to enhance the patient’s outcome by vascular assessment, multidisciplinary treatment, and infection control.

Supplementary Table S1 shows a heavy comorbidity burden, with neuropathy affecting most patients and contributing to the ulcer severity documented in Table 1. Microvascular and macrovascular complications, including retinopathy, proteinuria, heart failure, and stroke, reflect advanced systemic disease that limits physiological resilience. These patterns align with the higher rates of major amputation and mortality observed in Tables 6, 7. Supplementary Table S2 reports widespread moderate to severe infection, consistent with the osteomyelitis rates described in Table 1. Resistant organisms such as MRSA and ESBL strains created a challenging therapeutic environment that shaped the antibiotic and surgical strategies shown in Table 3. These infection characteristics underpin the strong predictive effect of infection severity on amputation outcomes in Table 6.

Supplementary Table S3 indicates pronounced systemic inflammation and metabolic stress, aligning with the sepsis burden in Table 1. Low albumin and elevated creatinine reinforce the high prevalence of CKD noted in Supplementary Table S1 and its impact on mortality risk in Table 7. These laboratory abnormalities help explain the greater need for intensive management and ICU care seen in Table 2. The patterns observed in tis cohort align with broader trends documented in the literature. The global burden of diabetic foot ulcers remains substantial, with high rates of infection, recurrent hospitalization, and amputation. A recent systematic review estimated that infection complicates up to 60% of ulcers and that ulcer-related hospitalizations impose a significant clinical-economic load on health systems (22, 23).

The strong association between PAD and amputation echoes existing data consistently shown that arterial insufficiency is one of the most potent risk factors for limb loss in diabetic foot disease (24). Moreover, infection severity, represented here by osteomyelitis and sepsis, was also a robust predictor in our cohort, consistent with findings from multicenter studies demonstrating that severe foot infection markedly increases the risk of surgical limb loss (25). Age and renal disease as predictors of mortality likewise reflect known biology. In our data, patients ≥ 65 or with CKD had significantly higher odds of death (Table 7). These factors have been reported in other cohorts: for example, older age, microvascular complications, and systemic comorbidities strongly predict adverse outcomes in diabetic foot disease (18, 26).

**Figure 6:**
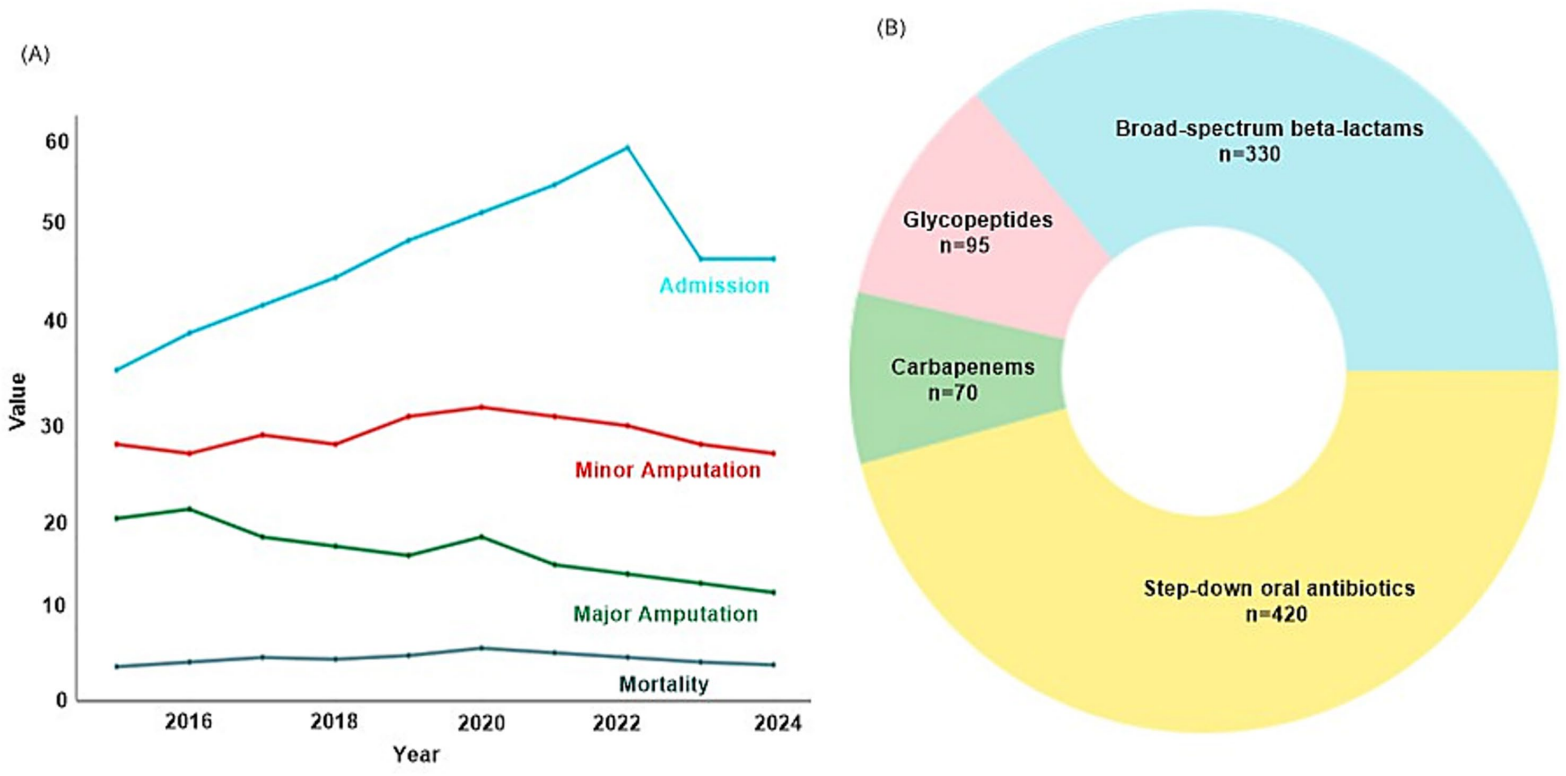
**(A)** Annual trends in diabetic foot admissions, minor amputations, and major amputations from 2015 to 2024. **(B)** Antibiotic utilization patterns in hospitalized diabetic foot patients, with broad-spectrum beta-lactams forming the most frequently used class.

Table 1 confirms that late presentation is typical, with high frequencies of Wagner 4–5 ulcers, PAD, and osteomyelitis producing an ischemic–infective phenotype that explains the extensive surgical workload reflected in Table 3. Hospital burden described in Table 2, including prolonged admissions and significant ICU demand, is consistent with international findings showing that diabetic foot disease remains one of the most resource-intensive complications of diabetes (8). The regression results in Tables 6, 7 confirm that major limb loss and mortality arise from interacting ischemic, infectious, and systemic factors rather than isolated clinical events. Finally, Table 4 and Figure 6A suggests modest improvements in major amputation rates over time, yet stable mortality and persistent readmissions imply that outpatient surveillance and early referral remain insufficient (27).

The results not only help identify known risk factors, but also give clinicians useful information for making decisions. The combination of advanced ulceration and peripheral arterial disease, osteomyelitis, and systemic infection identified a subset that would benefit from rapid multidisciplinary evaluation that involved a vascular surgeon, infectious disease, endocrinology, and wound management specialists. Prompt identification of this risk profile can allow timely intervention, best limb-salvage options and minimize extended hospital stays and rehospitalizations.

From a public-health perspective, these findings support several strategic priorities. First, earlier vascular assessment ideally at outpatient or community levels may identify PAD before critical limb ischemia develops. Given that PAD emerged as the strongest predictor of major amputation in our regression model (adjusted OR 4.15, Table 6), screening and referral pathways could have a transformative impact. Second, infection control must remain central: given the high prevalence of moderate-to-severe infection in our cohort (Supplementary Table S2), prompt initiation of empiric broad-spectrum antibiotics, followed by culture-directed therapy, is critical. This aligns with clinical guidance calling for early aggressive management of infected diabetic foot ulcers (28). Third, patients with CKD and advanced age represent a particularly fragile group; multidisciplinary teams, including nephrology, vascular surgery, infectious disease, and wound care, should be prioritized for integrated care (29–31).

The observed antibiotic usage pattern (Supplementary Table S8 and Figure 6B) highlights a multi-tiered approach to infection management in diabetic foot ulcers. The high prevalence of step-down oral antibiotics suggests favorable clinical responses enabling transition from intravenous to oral therapy and supports de-escalation strategies (32). The substantial use of broad-spectrum beta-lactams aligns with empiric protocols for polymicrobial and Gram-negative coverage. Lower utilization rates of glycopeptides and carbapenems likely mirror antimicrobial stewardship efforts and their indication for multidrug-resistant organisms. These trends underscore the importance of judicious antibiotic selection, local resistance patterns, and tailored therapy for optimizing infection outcomes in this patient group (21, 33).

Biomechanical and behavioral research reports give further context to the functional pathways related to the development of diabetic foot progression. The beneficial effect of shoe cushioning on lowering impact loading and muscle activation during unexpected landings has been demonstrated, and the effect is smaller during self-initiated movements (34). This indicates that there is a possibility that external mechanical factors can affect the stress responses of tissue and this can be relevant to the repetitive microtrauma in neuropathic feet. Likewise, changes in the properties of the sports surface during running change the distribution of load across the foot, suggesting that the properties of the ground interact directly with load distribution to the lower limb (35). Some long-term adaptations in the Achilles tendon have also been associated with retraining interventions and habitual foot strike patterns, as evidenced by changes in tendon mechanical loading and morphology after retraining (36, 37). In addition to mechanical factors, physical activity and patterns of activity and/or movement at home in the face of limited mobility have been linked to overall well-being and functional capacity, suggesting the systemic importance of levels of physical activity for maintaining musculoskeletal and metabolic health (38, 39). Together, these data support the notion that external loading conditions, gait behavior, and activity patterns can interactively contribute to tissue vulnerability and recovery potential in a patient with diabetic foot disease.

### 4.1 Strengths and limitations

This study has several important strengths. First, it includes a relatively large consecutive cohort of hospitalized patients with diabetic foot disease evaluated over a 10-year period, allowing comprehensive assessment of temporal trends in hospitalization burden, amputation patterns, and mortality. Second, the integration of demographic, microbiological, laboratory, vascular, and surgical variables enabled comprehensive risk stratification and multivariable evaluation of factors associated with adverse clinical outcomes. Unlike many previous studies that focused primarily on amputation risk, the present study simultaneously evaluated healthcare utilization, treatment characteristics, amputation outcomes, and short-term mortality within a single longitudinal cohort, providing a more comprehensive characterization of diabetic foot disease in routine clinical practice.

However, several limitations warrant consideration. The single-center design may limit generalizability to different health-care settings with variable access to vascular services. We lacked long-term follow-up data beyond 1 year for some patients, which constrains interpretation of survival beyond the immediate post-hospital period. While our multivariable models adjusted for major confounders, residual confounding is possible (e.g., unmeasured socioeconomic or behavioral factors). Finally, missing data, although limited, were handled via imputation, which could bias estimates if missingness was non-random (14, 21). Detailed severity grading of PAOD was unavailable for a subset of patients because of variations in historical documentation practices. Consequently, stratified analyses according to vascular disease severity could not be performed.

### 4.2 Future perspective

Policy actions supported by these data include establishing rapid-access local triage for foot ulcers, integrating ankle–brachial index or bedside perfusion testing into primary clinics, expanding community podiatry and diabetes education, and strengthening pathways for early revascularization. Future research should evaluate implementation strategies for multidisciplinary limb-salvage teams in resource-limited settings, examine cost-effectiveness of screening and early vascular referral, and assess the medium-term impact of pandemic-era service changes on amputation and survival rates (30, 40, 41). Prospective registries with standardized core datasets would improve comparability across centers and enable benchmarking of quality improvement initiatives. The IWGDF 2023 guidelines provide a contemporary implementation framework for many of these actions.

## 5 Conclusion

This study offers a detailed 10-year analysis of hospitalization burden, amputation trends and short-term mortality among the hospitalized patients with diabetic foot disease. The results are built into a longitudinal cohort and include clinical profiles related to adverse outcomes, combined with vascular status, severity of infection and clinical characteristics to provide a more comprehensive evidence base to inform multidisciplinary management of these patients and planning of healthcare resources. Severe ischemia, gangrene, renal impairment, poor metabolic control, and delayed surgical intervention was associated with higher rates of major amputation and mortality, although causal relationships cannot be established from the present study design. These findings support system-level investments in structured foot-care pathways, multidisciplinary limb-preservation teams, and accessible vascular evaluation to reduce preventable limb loss. Long-term population-based surveillance is essential to track evolving trends, evaluate service performance, and guide resource planning aimed at improving outcomes for individuals living with diabetic foot disease.

## Data Availability

The original contributions presented in the study are included in the article/Supplementary material, further inquiries can be directed to the corresponding author.
