# Supplementary material for: Hospital burden, amputation risk, and mortality trends in diabetic foot patients: a retrospective public health analysis
Source: Front Public Health. 2026 Jul 15;14:1753221. doi: 10.3389/fpubh.2026.1753221 (PMC13414974; doi:10.3389/fpubh.2026.1753221)
Supplement: Supplementary file 1 [file Table_1.DOCX]

**Hospital Burden, Amputation Risk, and Mortality Trends in Diabetic Foot Patients: A Retrospective Public Health Analysis**

**Table S1. Detailed Comorbidity Profile**

| **Comorbidity** | **n (%)** |
| --- | --- |
| Stroke history | 55 (11.0) |
| Heart failure | 70 (14.0) |
| Peripheral neuropathy | 415 (83.0) |
| Retinopathy | 230 (46.0) |
| Nephrotic-range proteinuria | 55 (11.0) |

**Table S2. Infection Severity and Microbiology**

| **Variable** | **Value** |
| --- | --- |
| Moderate infection | 240 (48.0) |
| Severe infection | 190 (38.0) |
| Culture-positive cases | 300 (60.0) |
| Polymicrobial infection | 160 (32.0) |
| MRSA | 95 (19.0) |
| ESBL organisms | 85 (17.0) |
|  |  |

**Table S3. Laboratory Parameters at Admission**

| **Variable** | **Mean ± SD or Median (IQR)** |
| --- | --- |
| WBC count (×10⁹/L) | 15.2 ± 5.8 |
| CRP (mg/L) | 112 (65–180) |
| ESR (mm/hr) | 73 (45–110) |
| Creatinine (mg/dL) | 1.9 ± 1.1 |
| Albumin (g/dL) | 3.1 ± 0.5 |

**Table S4. Detailed Breakdown of Minor vs Major Amputation Procedures**

| **Procedure Type** | **n (%)** |
| --- | --- |
| Toe amputation | 105 (21.0) |
| Ray amputation | 50 (10.0) |
| Transmetatarsal amputation | 35 (7.0) |
| Below-knee amputation | 70 (14.0) |
| Above-knee amputation | 20 (4.0) |

**Table S5. Readmission Analysis**

| **Variable** | **Value** |
| --- | --- |
| Readmission within 30 days, n (%) | 80 (16.0) |
| Primary cause of 30-day readmission | Infection (50%), wound breakdown (30%), uncontrolled diabetes (20%) |
| Readmission within 1 year, n (%) | 135 (27.0) |
| Most common reason | New ulcer or recurrent infection |

**Table S6. Comparison of Survivors vs Non-survivors**

| **Variable** | **Survivors (n = 455)** | **Non-survivors (n = 45)** | **p-value** |
| --- | --- | --- | --- |
| Age (years) | 56.5 ± 10.0 | 64.4 ± 9.5 | <0.001 |
| Sepsis, n (%) | 70 (15.4) | 25 (55.6) | <0.001 |
| CKD, n (%) | 135 (29.7) | 25 (55.6) | <0.001 |
| Major amputation, n (%) | 70 (15.4) | 20 (44.4) | <0.001 |

**Table S7. Time-to-Event (Mortality) Summary**

| **Variable** | **Value** |
| --- | --- |
| In-hospital mortality, n (%) | 35 (7.0) |
| 30-day mortality, n (%) | 45 (9.0) |
| Median time to death (days) | 12 (IQR 7–18) |

**Table S8. Antibiotic Use Patterns**

| **Antibiotic Class** | **n (%)** |
| --- | --- |
| Broad-spectrum beta-lactams | 330 (66.0) |
| Glycopeptides | 95 (19.0) |
| Carbapenems | 70 (14.0) |
| Step-down oral antibiotics | 420 (84.0) |
